# Nanodiamond Particles Reduce Oxidative Stress Induced by Methyl Viologen and High Light in the Green Alga *Chlamydomonas reinhardtii*

**DOI:** 10.3390/ijms24065615

**Published:** 2023-03-15

**Authors:** Taras K. Antal, Alena A. Volgusheva, Adil A. Baizhumanov, Galina P. Kukarskikh, Alessio Mezzi, Daniela Caschera, Gabriele Ciasca, Maya D. Lambreva

**Affiliations:** 1 Laboratory of Integrated Ecological Research, Pskov State University, 180000 Pskov, Russia; 2 Department of Biophysics, Faculty of Biology, Lomonosov Moscow State University, 119991 Moscow, Russia; 3 Institute for the Study of Nanostructured Materials, National Research Council, Monterotondo Stazione, 00015 Rome, Italy; 4 Dipartimento di Neuroscienze, Università Cattolica del Sacro Cuore, Fondazione Policlinico Universitario “A. Gemelli”, IRCCS, 00168 Rome, Italy; 5 Institute for Biological Systems, National Research Council, Monterotondo Stazione, 00015 Rome, Italy

**Keywords:** nanodiamonds, photosystem II, methyl viologen, high light, photoprotection, antioxidant activity, oxidative stress, photoinhibition

## Abstract

Widely used in biomedical and bioanalytical applications, the detonation nanodiamonds (NDs) are generally considered to be biocompatible and non-toxic to a wide range of eukaryotic cells. Due to their high susceptibility to chemical modifications, surface functionalisation is often used to tune the biocompatibility and antioxidant activity of the NDs. The response of photosynthetic microorganisms to redox-active NDs is still poorly understood and is the focus of the present study. The green microalga *Chlamydomonas reinhardtii* was used to assess the potential phytotoxicity and antioxidant activity of NDs hosting hydroxyl functional groups at concentrations of 5–80 μg NDs/mL. The photosynthetic capacity of microalgae was assessed by measuring the maximum quantum yield of PSII photochemistry and the light-saturated oxygen evolution rate, while oxidative stress was assessed by lipid peroxidation and ferric-reducing antioxidant capacity. We demonstrated that hydroxylated NDs might reduce cellular levels of oxidative stress, protect PSII photochemistry and facilitate the PSII repair under methyl viologen and high light associated stress conditions. Factors involved in this protection may include the low phytotoxicity of hydroxylated NDs in microalgae and their ability to accumulate in cells and scavenge reactive oxygen species. Our findings could pave the way for using hydroxylated NDs as antioxidants to improve cellular stability in algae-based biotechnological applications or semi-artificial photosynthetic systems.

## 1. Introduction

Detonation nanodiamonds (NDs) are particles obtained from the detonation soot of carbonaceous explosives in a low-cost and easily scalable process. They are characterised by small primary particle size (2–10 nm), a large surface area that is highly tunable, and the chemical inertness of the diamond core [1,2]. The tendency of the naturally hydrophobic ND particles to aggregate can be overcome by chemical modifications of the particle surface, which can reduce the grain size, improve their water solubility and biocompatibility, and enrich the physical and chemical properties of NDs [1,3,4,5]. The distinctive structural and chemical features of the NDs, their easy availability, and minimal toxicity, have enabled the development of numerous nanomaterial applications in biology and medicine, including drug delivery, sensing, imaging, implant coating, cancer treatment, etc. [1,3,6]. The use of detonation NDs in bio-related research has been further facilitated by the ability to obtain aqueous dispersed forms of these nanoparticles [7]. Despite being extensively studied for biomedical and bioanalytical applications in animal and human cell lines, little is known about the effects of NDs on photosynthetic organisms, including photosynthetic microbes, which motivated this study.

Microalgae are an important model system in both fundamental and application-oriented photosynthesis research [8,9,10,11]. Apart from being protagonists in studies aimed at elucidating the regulation and acclimation mechanisms of photosynthetic reactions, microalgae are emerging as a major biosource of biomass and high-value compounds well suited to various industrial needs [12,13]. However, the involvement of nanomaterials and nanotechnology in the development of algal biotechnological applications is still in its early stages [11,14,15]. In this regard, the potential effects of ND particles on the growth, photosynthetic activity, and production capacity of microalgae under favourable or stressful conditions could be of great concern for the advancement of algal biotechnology.

In general, particle interactions with biological materials at the nanometre scale, including both beneficial and adverse effects, are size, composition, and concentration-driven phenomena and depend on the target biospecimen [2,11]. Raw NDs failed to induce toxic effects in neuroblastoma cells and macrophages at concentrations of 25–100 μg/mL, unlike the other carbon allotropes used in the same study, such as carbon black, single-walled and multi-walled carbon nanotubes [2]. Similar high biocompatibility, lack of toxicity, and no generation of reactive oxygen species (ROS) have also been observed for NDs with different (-COOH, -COONa, -SO_3_Na) functional groups [16] and for hydroxylated NDs [17] in human cell lines, and carboxylated NDs in yeast cells [18]. Most of the scientific literature dealing with ND bio-applications confirms that the NDs are highly compatible with biomolecules and are not toxic to eukaryotic cells.

Various studies have also shown the antibacterial activity and toxicity of NDs in animal cells, which in most cases has been associated with the particle’s ability to induce oxidative stress. Adverse effects on cellular redox balance and viability were observed in Gram-positive and -negative bacteria exposed to oxidised NDs [19,20] and oxidised NDs containing anhydride groups [21]. Although the topic is still under investigation, ND toxicity is mainly related to the degree of surface oxidation of the particle, with excessive oxidative functionalisation increasing the ability of the nanomaterial to induce toxic effects. Individual studies have also shown a relationship between ND toxicity and particle size, although the effects obtained were not always consistent [19,22].

The ND surface chemistry is also responsible for the ability of the nanoparticles to promote the reduction of oxygen species [23]. The antioxidant activity and radical scavenging potential of NDs have been attributed to the numerous functional groups (mainly -OH and -COOH) and abundant C=C bonds on the ND surface [4,18,24,25]. The degree of UV-mediated lipid peroxidation and consequent aldehyde formation in soybean oil enriched with hydroxylated NDs was lower than in pure oil or oil enriched with non-modified nanoparticles [25]. The radical scavenging ability of hydrated carboxylated NDs was demonstrated in red blood cells and water under γ-irradiation [4]. Moreover, carboxylated NDs improved cell viability and lowered ROS accumulation in yeast cells under oxidative stress induced by H_2_O_2_ addition [18]. A growing number of studies are currently focusing on detonation NDs as long-lasting and stable antioxidants, which further expands the field of material applications towards cosmetics and smart packaging [26,27,28].

Here, we present the first study on the phenotypic response of the model green alga *Chlamydomonas reinhardtii* to oxidised detonation NDs under conditions of oxidative stress. Chloroplast ROS production is not limited to microalgae under unfavourable stress conditions. It is part of the routine functioning of plant photochemistry and, if left uncontrolled, can severely compromise photosynthetic energy conversion efficiency [29]. By introducing NDs close to the site of ROS generation, the free radicals can be scavenged before they damage nearby lipids, pigments, and photosynthetic reaction centre proteins, thus compromising the photosynthetic performance of the organism. To the best of our knowledge, all studies on ND interactions with microalgae are limited to the two research articles recently published by Zhang and colleagues [30,31]. The authors studied the 24 and 48 h response of the green alga *Chlorella pyrenoidosa* to oxidised and graphitised NDs at concentrations of 5–50 μg/mL, using phenotypic and transcriptional analyses. Microalgal growth was inhibited by all ND treatments, while the activity of antioxidant enzymes and lipid peroxidation were markedly altered by 50 μg NDs/mL [31]. Zhang and colleagues [30] have also demonstrated that the oxidation rather than graphitisation of the NDs can mitigate the negative effect of the nanoparticles on the *C. pyrenoidosa* cells. The algal cultures supplemented with oxidised NDs showed reduced growth inhibition and a low degree of organelle damage and oxidative stress compared to cells exposed to graphitised NDs. Surprisingly, in both studies, the photosynthetic machinery was not severely affected by the ND treatments, as evidenced by negligible changes in photosystem II (PSII) function and stimulation of photosynthetic pigment accumulation.

This study aimed to investigate the ability of oxidised detonation NDs to mitigate the adverse effects of oxidative stress conditions on the photosynthetic activity of the model green microalga *C. reinhardtii*. The surface chemistry of the NDs has been analysed in detail to relate the physiological responses observed in the algae to the structural characteristics of the nanomaterial. Thus, the toxicity of aqueous suspensions of detonation NDs, purified and functionalised with hydroxyl groups (hNDs), on microalgae was also evaluated. We demonstrated that hydroxylated NDs might reduce cellular levels of oxidative stress, protect PSII photochemistry, and facilitate PSII repair under methyl viologen (MV) and high light (HL) associated stress conditions. Our results showed that hNDs are less toxic to *Chlamydomonas* than other carbon-based nanomaterials, such as single-walled carbon nanotubes. We propose to exploit the antioxidant activity of hNDs to improve cell stress tolerance in the development of algal-based biotechnological applications or semi-artificial photosynthetic systems.

## 2. Results

### 2.1. Characterisation of the Nanomaterial

**Figure 1:**
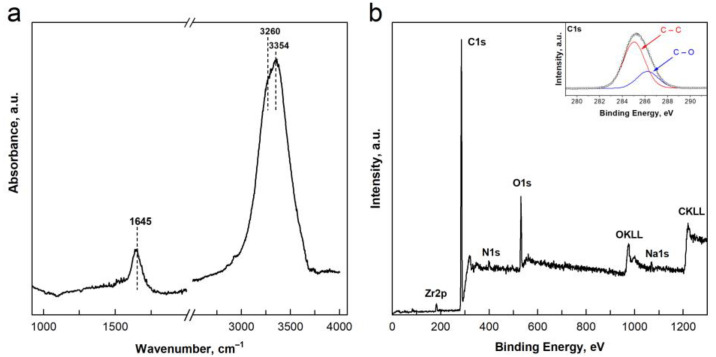
Characterisation of the surface chemical composition of hydroxylated ND particles. (**a**) FT-IR spectrum of hNDs in the 1000–4000 cm^−1^ range. (**b**) XPS survey scan spectrum of hNDs and the high-energy resolution C1s spectrum (insert).

In this study, we used detonation ND particles that were further refined and oxidised by the producer to obtain stable water suspension of negatively charged particles (see Section 4.1 of Materials and Methods). Fourier-transform infrared (FT-IR) spectroscopy was used to assess the surface functionalisation of the NDs. FTIR spectrum of NDs (Figure 1a) showed two main features related to the hydroxylation of the particle surface (bands in the 1600–1650 cm^−1^ and 3200–3500 cm^−1^ regions). The IR bands originating from OH bending modes in the 1600–1650 cm^−1^ region depend on the interaction of ND-OH groups with other functional groups on the particle surface or with water molecules surrounding the NDs. The signal peaking at 1645 cm^−1^ indicates the presence of strong hydrogen-bonded OH groups on the ND surface [32]. The signal in the range of 3200–3500 cm^−1^ is assigned to O-H stretching and can be attributed either to water molecules adsorbed on the NDs or to hydroxyl groups present on their surface. Both the broad peak at 3354 cm^−1^ and the shoulder at 3260 cm^−1^ could be attributed to strong intermolecular bonding with water molecules [33]. The absence of bands characteristic of the carbonyl stretching modes in the 1700–1800 cm^−1^ region can be related to the presence of functional groups with a low degree of oxidation [34].

The FT-IR spectroscopy indicated NDs with strong surface hydroxylation, which may alter the reactivity of the particles in an aquatic environment. In the rest of the text, the nanomaterial is referred to as hydroxylated NDs or hNDs.

The surface chemical composition of hNDs particles was characterised by the presence of carbon and oxygen atoms, with atomic concentrations of ~87 at.% and 10 at.%, respectively (Figure 1b and Supplementary Table S1). The XPS analysis also revealed the presence of other elements, such as Zr, N, and Na, which may originate from the post-synthesis procedures used to de-aggregate, purify, and functionalise the particles [1]. These elements were only present in trace amounts, as their total atomic concentration did not exceed 3 at.%. Peak-fitting analysis of the high-energy resolution spectrum of the C1s peak revealed the presence of two components in the C1s signal, assigned to C-C and C-O bonds, characterised by a binding energy (BE) of 285.0 eV and 286.6 eV, respectively (Figure 1b, inset). The contribution of the C-O signal was quantified at ~14 at.% of the sample atomic concentration. Based on the FT-IR analyses, it is reasonable to confirm the presence of C-OH functional groups on the hND surface. The characterisation of the degree of carbon hybridisation (sp^2^ or sp^3^) of the sample showed a D value of 12.8 eV, which is typical for C 100% hybridised in C sp^3^ (diamond) [35], and a high degree of purity of the NDs.

**Figure 2:**
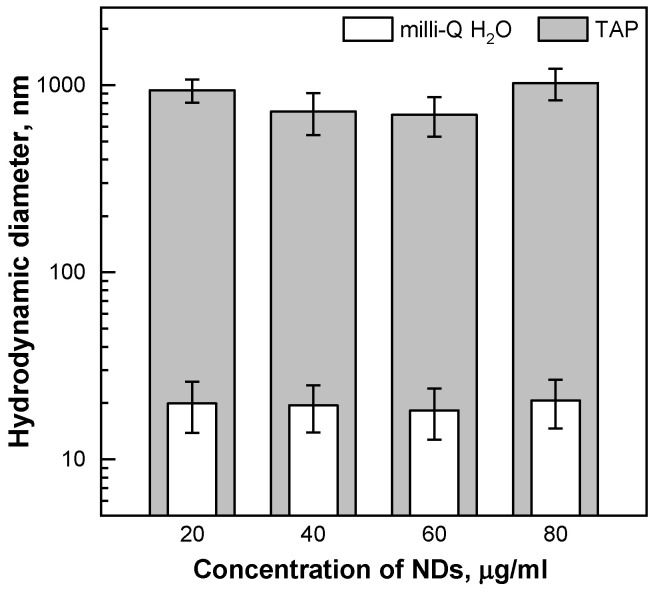
hNDs size distribution (hydrodynamic diameter) as a function of particle concentration in milli-Q water (white bars) and in TAP medium (grey bars). For clarity, the data are visualised on a logarithmic *y*-scale. Average values of 5 independent measurements (±SD, *n* = 5).

Changes in the average hydrodynamic diameter of the hydroxylated NDs were studied as a function of particle concentration, in the range from 20 to 80 μg hNDs/mL, in milli-Q water and in TAP medium (Tris-Acetate-Phosphate buffer used to grow algae). The hND colloidal suspension was stable in water (Figure 2), with a mean hydrodynamic diameter of ~20 nm and a negative surface charge of circa −35 mV. The value of both parameters was similar to the nanoparticle characteristics claimed by the manufacturer. A rapid increase in the particle size was observed when hNDs were added to the TAP medium, with the formation of aggregates ranging in size from a few hundred nanometres to one micrometre (Figure 2). In the presence of electrolytes, the surface charge of the nanoparticles was neutralised. These results are consistent with literature data on the stability of NDs in a solution of different salts [36]. It appears that attractive van der Waals forces and particle agglomeration dominate, as the total concentration of salts in the TAP medium exceeds the critical coagulation concentration.

### 2.2. hND Phytotoxicity in C. reinhardtii Cells

**Figure 3:**
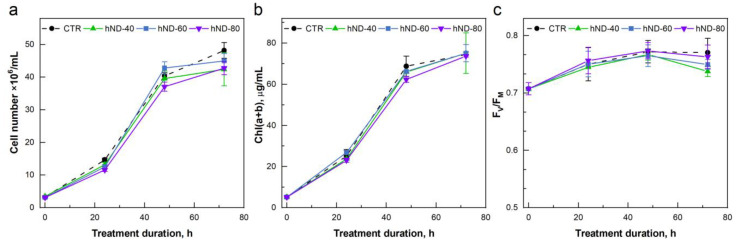
Long-term toxicity test of high hND concentrations in *C. reinhardtii* cells. Algal cultures containing 5.1 ± 0.3 µg Chl/mL were supplemented with 40, 60, or 80 µg hNDs/mL (hND-40, hND-60 and hND-80) and the changes in cell proliferation (**a**), chlorophyll content (**b**) and F_V_/F_M_ (**c**) were monitored for 72 h. Average values of 2 independent experiments with 3 biological replicates (±SD, *n* = 6).

NDs are generally regarded as biocompatible and cell-penetrable particles investigated for intracellular delivery, bioimaging, and tissue engineering [1]. However, some studies have highlighted the pro-oxidant activity or cytotoxicity of NDs, depending on the biological sample and the concentration, purity, and functionalisation of the nanomaterial [2]. Literature on the interaction of functionalised or pristine NDs with algal cultures is limited to very few investigations [30,31]. In the present study, low concentrations of hydroxylated NDs (5, 10 and 20 µg/mL) showed no phytotoxic effects on *Chlamydomonas* cells in either the short-term (5 h, Supplementary Figure S1a) or long-term (72 h, Supplementary Figure S2) experiments. In the short-term toxicity tests, the high-concentration treatments (40, 60, and 80 µg hNDs/mL) did not induce changes in the F_V_/F_M_ parameter related to the maximum quantum yield of the photosystem II (PSII) photochemistry (Supplementary Figure S1b). The high hND concentrations in the long-term tests altered the growth and Chl of the cultures already at the T24 point (24 h of treatment, Figure 3a,b), whereas the reduction in the PSII photochemistry was not pronounced until the end of the treatment (T72—72 h of treatment, Figure 3c). The negative effects (Figure 3a,b) were evident in the hND-60 and hND-80 samples, where the addition of hNDs also provoked cell flocking. Most probably, the delay in culture development was related to the entrapment of cells in agglomerates, but the physiological changes were mild enough and were not accompanied by a significant reduction in PSII photochemistry during the first 48 h.

### 2.3. hND-Mediated Protection of C. reinhardtii Cells against Oxidative Stress Induced by MV

Methyl viologen molecules easily cross the cell wall and membranes of plant cells and enter the chloroplast compartment. In the chloroplast, MV acts as an alternative electron acceptor, extracting electrons from the Fe-S proteins of photosystem I and generating superoxide radicals (O_2_^•−^) [37,38]. In light-exposed cells, the electrons deriving from the photosynthetic electron transport chain (ETC) continuously reduce the MV from a divalent to a monovalent cationic state. The latter is rapidly reoxidised by O_2_ forming O_2_^•−^, which initiates the generation of other ROS. Thus, MV interferes with the photosynthetic electron flow by inducing oxidative damage to photosynthetic pigment–protein complexes, in particular on PSII, resulting in the inhibition of photosynthetic activity. The latter can be detected by measuring the decrease in PSII photochemistry, expressed as F_V_/F_M_ [39].

The ability of hydroxylated NDs to protect microalgae from oxidative stress was tested for a wide range of nanoparticle concentrations (5 ÷ 80 µg/mL) using two different sets of experiments, termed “immediate effect” and “24 h pre-incubation effect”. In the first case, the MV and hNDs were added simultaneously to the cultures; in the second case, cells were pre-incubated with hNDs for 24 h, resuspended in a nanoparticle-free TAP medium and exposed to MV. In all experiments, we used a concentration of 3 µM MV, which induced an approximately 50% decrease in the F_V_/F_M_ parameter (see Section 4.5.1 of Materials and Methods).

**Figure 4:**
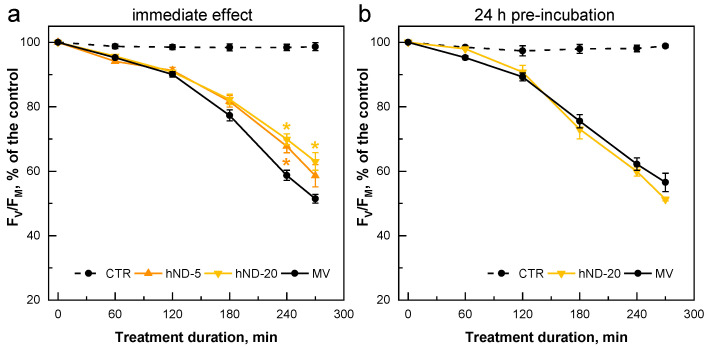
Changes in the F_V_/F_M_ parameter induced by 3 µM MV in *C. reinhardtii* cultures supplemented with a low concentration of hNDs (5 and 20 µg/mL). (**a**) “immediate effect”—MV and hNDs were added simultaneously to the cultures. (**b**) “24 h pre-incubation effect”—MV was added to cultures pre-incubated for 24 h with hNDs. Values are expressed as a % of the control, F_V_/F_M_(T0) = 0.73 ± 0.005 (**a**) and F_V_/F_M_(T0) = 0.74 ± 0.006 (**b**). Asterisks indicate statistically significant differences between the MV and hND samples at corresponding time points, *p* < 0.05. Means of 2–4 independent experiments with 2–3 biological replicates (±SE, *n* = 4–12).

**Figure 5:**
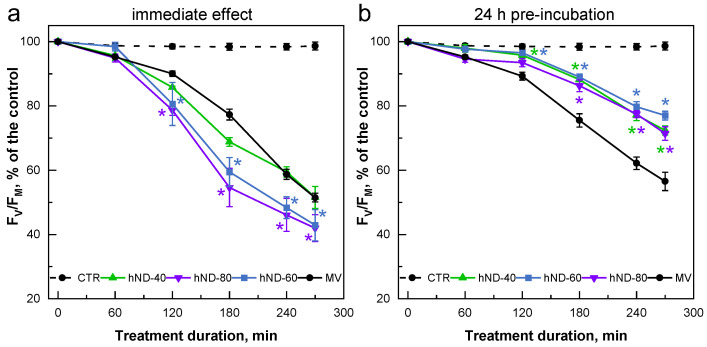
Changes in the F_V_/F_M_ parameter induced by 3 µM MV in *C. reinhardtii* cultures supplemented with a high concentration of hNDs (40, 60 and 80 µg/mL). (**a**) “immediate effect”—MV and hNDs were added simultaneously to the cultures. (**b**) “24 h pre-incubation effect”—MV was added to the cultures pre-incubated for 24 h with hNDs. Values are expressed as a % of the control, F_V_/F_M_(T0) = 0.73 ± 0.008 (**a**) and F_V_/F_M_(T0) = 0.73 ± 0.004 (**b**). Asterisks indicate statistically significant differences between the MV and hND samples at corresponding time points, *p* < 0.05. Means of 2–3 independent experiments with 3 biological replicates (±SE, *n* = 6–9).

In the two sets of experiments, the pattern of MV-mediated reduction of the F_V_/F_M_ parameter was quite different in the low (Figure 4) and high (Figure 5) hND concentration ranges. Low hND concentrations (5 and 20 µg/mL) increased F_V_/F_M_ by 10–12% when MV and hNDs were simultaneously added to the culture (immediate effect, Figure 4a). The preincubation with 20 µg hNDs/mL for 24 h did not provide any benefit to the algal cultures in response to the subsequent treatment with MV (Figure 4b).

On the contrary, the presence of high hND concentrations enhanced the MV-induced inhibition of PSII photochemical activity in a concentration-dependent manner, resulting in up to 25% greater reduction in the F_V_/F_M_ in hND-80 compared to the sample treated with MV alone (immediate effect, Figure 5a). This effect was statistically significant in the hND-60 and hND-80 samples, where algal cells entrapped in agglomerates were visible as a greyish deposit on the flask walls. However, it is difficult to attribute the observed change in the F_V_/F_M_ to cell agglomeration or the possible phytotoxicity of high hND concentrations only since 5 h incubation with 40, 60, and 80 µg hNDs/mL alone did not significantly alter the F_V_/F_M_ parameter (Supplementary Figure S1b). Nevertheless, it is possible that the presence of hNDs further enhances the negative effects of MV-induced oxidative stress on cell fitness. This effect was not apparent in the hND-5 and hND-20 samples.

In response to the subsequent MV treatment, the 24 h pre-incubation with high concentrations of hNDs resulted in well-developed protection of the PSII photochemical activity. The F_V_/F_M_ values of the hND-pretreated cells were 15 to 20% higher than those of the MV-exposed control samples (Figure 5b). The effect of the hND pre-incubation was significant after the first 2 h (in hND-40 and hND-60) and 3 h (also in hND-80) of the exposure to MV. Interestingly, hND-associated PSII protection did not show any correlation with the particle concentration used in the pre-incubation procedure.

All cultures in the “24 h pre-incubation effect” experiments were analysed for culture development and PSII photochemistry before being exposed to MV treatment (Supplementary Figure S3). The results showed that the 24 h incubation with hNDs had no effect on PSII photochemistry in any of the treatments. However, the cell proliferation (Supplementary Figure S3b) and Chl content of the samples (Supplementary Figure S3c) were significantly reduced at hND concentrations higher than 40 µg/mL. This observation and the lack of apparent advantage of using concentrations higher than 40 µg hNDs/mL (Figure 5b) motivated the choice of the hND-40 treatment in the remaining experiments. Furthermore, the “24 h pre-incubation effect” experimental set-up was favoured in order to exclude the occurrence of non-specific reactions involving the hNDs using algal cells resuspended in a medium free of hNDs.

The degree of MV-induced oxidative stress in hND-40 samples, in which hND pre-incubation showed a clear protective effect, alleviating the F_V_/F_M_ decrease (Figure 5b), was evaluated by assessing malondialdehyde (MDA) content and ferric-reducing antioxidant power (FRAP) in the cells. MDA and FRAP were used as indicators of lipid peroxidation (membrane integrity) and non-enzymatic antioxidant capacity of the cells, respectively [40,41]. Table 1 shows the MDA and FRAP of CTR and hND-40 samples at the T0 point (before MV addition) and after 3 h of incubation with 3 µM MV under growth PPFD (T3). At the T0 point, both control and hND-pretreated cultures showed relatively low levels of MDA (1.7–1.8 µmol/g DW) and FRAP (0.2 mmol Fe(II)eq/g DW), which were consistent with those found in the algal cells under optimal growth conditions [41]. Exposure of cells to MV resulted in a significant increase in both oxidative stress markers, but hND-pretreated cultures showed lower MDA and FRAP levels at T3 compared to the control cells. In the cultures pre-treated with hNDs, the MDA and FRAP values increased by 53% and 55%, respectively, compared to the 72% and 80% increase in the control samples. This result supports the notion that the observed hND-induced protection of PSII photochemistry in the presence of MV (Figure 5b) may be associated with a corresponding decrease in the level of oxidative stress in the cells.

### 2.4. hND-Mediated Protection of C. reinhardtii Cells under High Light Conditions

**Figure 6:**
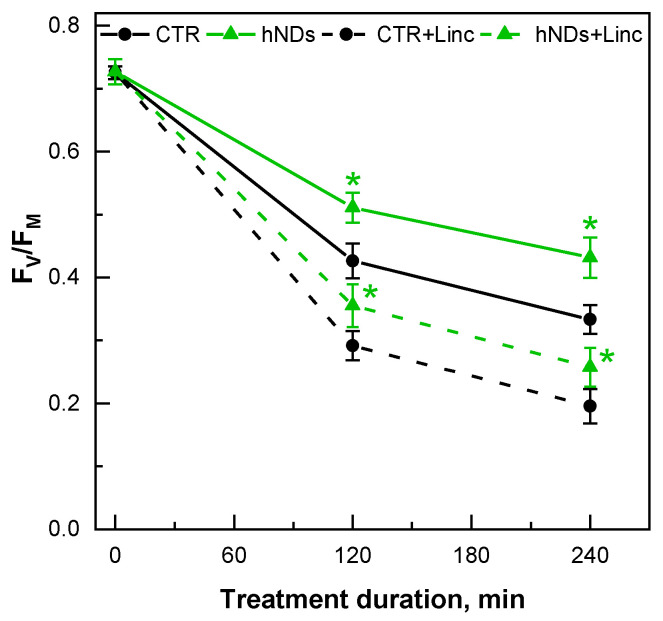
Changes in the F_V_/F_M_ of *C. reinhardtii* cultures pre-incubated with 40 µg hNDs/mL for 24 h and control cells exposed to a PPFD of 1000 µmol/m^2^/s for 4 h. Where indicated, 400 μg Linc/mL was added at the start of the HL treatment. Asterisks indicate statistically significant differences between the CTR and hND samples at corresponding time points at *p* < 0.05. Means of 2 independent experiments with 3 biological replicates (±SD, *n* = 6).

Exposure of the algal cells to supraoptimal light conditions (high light, HL) is often used to induce photoinhibition in photosynthetic samples [42,43]. Photoinhibition refers to the reduction of PSII activity under strong light, where the mechanisms include damage to PSII and the PSII repair cycle by reactive oxygen species (mainly singlet O_2_) generated under a high light [39,42]. Therefore, we tested the ability of *Chlamydomonas* cells pre-incubated with 40 µg hNDs/mL to withstand HL-induced photoinhibitory and oxidative stress conditions by exposing the cultures to a PPFD of 1000 µmol/m^2^/s for a period of 4 h (Figure 6). This light intensity induced approximately 50% inhibition of PSII activity for the duration of the experiment (see Section 4.5.2 of Materials and Methods). To distinguish between direct PSII photodamage and PSII repair processes during HL treatment, some experiments were performed in the presence of lincomycin (Linc), an inhibitor of plastid protein synthesis that blocks PSII repair [44].

In the control cells, the exposure to HL almost halved the F_V_/F_M_ values (41% reduction) during the first 2 h, followed by a lower decrease of a further 14% until the end of the experiment. Changes in the F_V_/F_M_ induced by HL in the hND samples followed a pattern similar to that of controls, except that the total reduction in PSII photochemistry did not exceed 41% across the experimental time. These results suggest that hNDs may protect PSII from photodamage to a similar extent as was observed when PSII damage was induced by oxidative stress in the presence of MV.

The addition of Linc did not alter the shape of the F_V_/F_M_ dynamics, and as expected, inhibition of the PSII repair cycle resulted in a greater decrease in the F_V_/F_M_ parameter (Figure 6, dashed lines). In the cultures pre-incubated with hNDs, the photoinhibition was attenuated, and at the end of the experiment, the F_V_/F_M_ parameter was reduced by 64% compared to the 73% reduction in the control. The hND-mediated protection on PSII photochemistry (F_V_/F_M_) under HL conditions was lowered from 14 % (∆% inhibition (NDs—CTR) at the T4 point (4 h of treatments)) in the absence of PSII repair inhibitor to ~8% in the presence of Linc. The decrease in the apparent protection of PSII photochemistry mediated by hNDs in the presence of Linc supports the idea that hNDs are involved in the stimulation/protection of PSII repair processes in addition to the reduction of direct damage to PSII.

The protection of photosynthetic reactions mediated by hydroxylated NDs under HL conditions was also confirmed by measurements of the light-saturated O_2_ evolution rate of algal cells exposed for 2 h to a PPFD of 1000 µmol/m^2^/s (Table 2). HL treatment significantly reduced the O_2_ production rate of both the control and hND-preincubated cells. The photosynthetic capacity of the hND samples was reduced by 29% compared to the 43% reduction in the control. HL exposure provoked a marked increase in the dark respiration rate (122% in the CTR) that was partially overcome in the hND-preincubated cultures, where the increase was reduced to 94%. The elevated respiration rate observed under HL conditions was most likely due to the acceleration of chlororespiration, which acts as an alternative electron transport pathway, directing the excess of electrons towards molecular oxygen in the chloroplast [10,45].

HL-induced oxidative stress was also evaluated by measuring MDA and FRAP levels and recording the capacity of the cells to generate singlet oxygen (^1^O_2_) at a PPDF of 3000 µmol/m^2^/s (Table 3)—two hours of HL altered the oxidative stress indicators to a lesser extent in comparison with the MV treatment (Table 1). However, HL treatment still triggered an increase in MDA and FRP levels, which did not exceed 28% on average for all variants. At the T0 point (before the HL treatment), the control cells generated ^1^O_2_ at a rate of 16 ± 3 µmol ^1^O_2_/mg Chl/h, whereas hND-preincubated cultures showed a similar yet slightly lower value (12 ± 2 µmol ^1^O_2_/mg Chl/h). After 2 h of HL (T2 point), the rate of histidine-assisted ^1^O_2_ production was increased to a similar extent (more than 60%) in both the control and hND-preincubated cells. In general, hND pre-incubation did not prove beneficial in reducing oxidative stress levels under HL.

It is noteworthy that 2 h of HL induced an approximately two-fold greater reduction in F_V_/F_M_ compared to MV, whereas the HL-induced effects on oxidative markers were less pronounced than in the MV treatment. These observations are consistent with the notion that HL-associated oxidative stress is primarily related to the generation of singlet O_2_, which is a short-lived ROS with effects mainly limited to PSII reaction centre proteins.

## 3. Discussion

Deliberately functionalised ND particles have been extensively studied as components with long-lasting antioxidant activity and ROS scavenging properties for various applications ranging from cosmetics [24] to food preservation and smart packaging production [25,26,27]. Nevertheless, the ability of NDs to promote ROS reduction in photosynthetic microorganisms is still an unexplored area and was the primary focus of this study.

Our data clearly demonstrated the capacity of hydroxylated NDs to protect the PSII photochemistry and reduce the levels of oxidative stress in the model microalga *C. reinhardtii* under oxidative stress conditions induced by either MV or HL treatment. Being continuously supplied with electrons by the photosynthetic ETC, the MV molecules produce superoxide radicals at the level of PSI that can originate other ROS in the chloroplast [37]. The HL-associated stress is a more complex phenomenon involving, among other things, the generation of single oxygen at the level of PSII [42,43]. ROS accumulation in the chloroplast is directly related to damage to membrane lipids, proteins and, in particular, the photosynthetic pigment-protein complexes. PSII is one of the primary targets of oxidative stress that can severely compromise its function. In our study, the ability of hydroxylated NDs to alleviate the impairment of PSII photochemistry (Chl *a* fluorescence parameter F_V_/F_M_) under oxidative stress was demonstrated by the particle-mediated reduction of PSII inhibition (Figure 5b and Figure 6). In addition, the hND-treated samples showed a lower increase in the dark respiration rate (rather due to chlororespiration) and maintained higher O_2_ evolution rates than the control cells under the HL conditions (Table 2). In all cases, the hND-treated cultures showed lower levels of oxidative stress markers compared to the corresponding controls, especially in the MV experiments (MDA and FRAP, Table 1 and Table 3). Most likely, the mechanism of the observed hND-mediated protection is related to the mitigation of oxidative stress through ROS quenching and free radical scavenging. A similar mode of action has been proposed in various studies demonstrating the radical scavenging and antioxidant activity of differently functionalised NDs (mainly via mild oxidation) in red blood cells [4], soybean oil [25,26], yeast cells [18], in sunscreen formulations [24], among others.

The ability of the NDs to quench ROS and scavenge free radicals is usually linked to their surface chemistry [23,24,25]. In fact, it is demonstrated that ND functionalisation can improve the antioxidant properties of the particles and contribute to their biocompatibility and water solubility [1,4]. Conversely, a high degree of oxidation of NDs can release the pro-oxidant activity and cytotoxicity of the NDs [2,19,20]. A relationship has been found between the type of molecules bound to the ND surface and the particle redox activity, with the latter decreasing in the order ND-CH_2_-NH_2_ > ND-CH_2_-OH > ND-COOH [46]. Thus, the antioxidant activity of the hNDs observed in the present study may be related to the presence of the C-OH with a total atomic concentration of ~14 at.%. This suggestion agrees with the reduced levels of oxidative stress observed in soybean oil under UV exposure [25] and in red blood cells under γ-irradiation [4] supplemented with hydroxylated and hydrated carboxylated NDs, respectively.

Notably, significant hND-mediated protection on PSII photochemistry (~20% and ~14% in MV and HL treatments, respectively) was observed only in the cultures pre-incubated with nanoparticles (Figure 5b and Figure 6). When the cells were exposed to MV in the presence of “free” hNDs in the medium, PSII photochemistry was inhibited by up to 25% (Figure 5a, hND-60 and hND-80). According to our results, a direct negative effect of hNDs on PSII seems very unlikely since the nanoparticles used in this study did not show any obvious negative effects on the PSII activity under optimal growth conditions (Figure 3 and Supplementary Figure S3). Indeed, PSII photochemistry appeared to be the least affected parameter in the phytotoxicity tests we performed. In the 24 h pre-incubation experiments (Supplementary Figure S3), the F_V_/F_M_ values remained unchanged in all hND treatments, although the culture growth and Chl content were reduced in hND-60 and hND-80 samples. A similar stability of the F_V_/F_M_ parameter, accompanied by growth inhibition, was also observed in the green alga, *C. pyrenoidosa*, treated for 48 h with 50 µg/mL of oxidised NDs [30]. Assuming that PSII photochemistry is hardly altered by the hNDs in healthy control cells, it is still possible that the nanoparticles could aggravate the PSII inhibition under conditions of oxidative stress where the protection and repair mechanisms are already compromised. We are inclined to believe that the increase in photoinhibition in the “immediate effect” at high hND concentrations could also be related to the entrapment of cells in aggregates and the colloidal stability of hNDs in the TAP medium (Figure 2). The scavenging potential of the functionalised NDs depends on the surface area available for their interactions with the ROS, and particle aggregation may play an essential role in limiting their antioxidant activity [4]. Nevertheless, a direct redox interaction between hNDs and MV molecules in the medium, leading to the generation of additional ROS in the sample media, could not be excluded.

The similar degree of protection against MV-mediated oxidative stress among the samples pre-incubated with 40, 60, and 80 µg hNDs/mL (Figure 5b) indicated no relation between the relative amount of active sites inside the cells and the hND concentration of the treatment. This effect could be related to the concentration-dependent limitation of particle internalisation and/or the hND colloidal stability in the TAP medium. Such analyses are beyond the scope of the present research and should be a subject of an independent survey. However, due to the nature of the hND-mediated protection observed in this study, it is reasonable to hypothesise that a certain amount of hNDs manages to enter the cells during the 24 h pre-incubation. This could explain not only the hND-mediated protection against direct PSII damage and the effect on membrane lipid peroxidation and antioxidant capacity of the cells but also the positive effect of the hNDs on the PSII repair cycle. The contribution of the hNDs to the mitigation of the direct light-induced damage on PSII and to its repair was suggested by data obtained in the presence of Linc, a substance that inhibits the plastid synthesis of proteins [44]. The degree of hND-mediated “protection” on PSII under HL was higher in the Linc-free samples compared to the Linc-containing cells (Figure 6). It has been shown that ROS accumulation in the chloroplasts can interfere with the normal functioning of PSII and significantly hinder PSII repair [47]. Thus, the hNDs may contribute to the stimulation/protection of PSII recovery by reducing the level of ROS in the chloroplast. This finding is consistent with the proposed mode of action of the functionalised via oxidation NDs based on their involvement in ROS scavenging and reduction of oxidative stress in the cell.

Our results highlighted the low sensitivity of PSII photochemistry in *C. reinhardtii* to hydroxylated NDs under physiological conditions. This observation generally agrees with the results of Zhang and colleagues [30,31], who came to similar stability of PSII-related parameters. However, these authors calculated an EC_50_ value of ~26 μg/mL for the unmodified NDs and observed a significant reduction in cell number at 5 μg NDs/mL for both oxidised and graphitised nanoparticles [30,31]. In our study, hydroxylated NDs were less toxic and altered algal growth only at concentrations higher than 40 μg hNDs/mL. Furthermore, in contrast to the antioxidant activity we have demonstrated, Zhang and colleagues [30,31] observed ND-mediated damage on cell organelles and an increase in antioxidant enzyme activity and MDA levels. To some extent, these discrepancies may be related to the different sensitivity of *Chlamydomonas* and *Chlorella* to engineered ND nanoparticles. However, given the key role of ND surface chemistry in particle interactions, it is more reasonable to assume that the different behaviour of the NDs was due to differences in the amount and chemical nature of the functional groups present on the particle surface. An excessive functionalisation or the lack of surface modifications has often been related to the reduction in the biocompatibility and increase in pro-oxidant activity of the NDs and enhanced toxicity [4,19,21].

In conclusion, this study provides the first evidence of the ability of hydroxylated NDs to reduce cellular levels of oxidative stress, protect PSII photochemistry, and facilitate PSII repair under MV- and HL-associated stress conditions. hNDs were found to be less toxic to microalgae than single-walled carbon nanotubes [15,48], similar to the higher biocompatibility of NDs compared to other carbon nanomaterials that have been demonstrated in human cells [2]. Although there is no direct evidence of cellular internalisation of the nanoparticles, the nature of the hND-mediated effects observed in this study suggests that the particles are localised inside the cells. Future analyses should focus on elucidating the cell internalisation and localisation dynamics of NDs in relation to particle functionalisation. The possibility of tuning the antioxidant activity of NDs, and eventually also their toxicity, in microalgae cells by ad hoc functionalisation should also be explored. Furthermore, the research could be extended by evaluating the antioxidant effects of NDs under different stress conditions, such as nutrient deprivation, anaerobiosis, and low and high temperature, which are often used to modulate algal metabolism towards the production of specific compounds of interest. Our findings could pave the way for using functionalised NDs as antioxidants to improve cellular stability in algae-based biotechnological applications or semi-artificial photosynthetic systems.

## 4. Materials and Methods

### 4.1. Properties and Characterisation of the Nanomaterial

The nanodiamonds (ND5nmNH2O) used in the present study were synthesised by detonation and were purchased from Adámas Nanotechnologies Inc. (Brilliant Diamond Solutions, Raleigh, NC, USA, www.adamasnano.com (accessed on 12 March 2023)). As claimed by the producer, the detonation product was further refined and oxidised to obtain water suspensions of purified nanodiamonds containing particles with 5–10 nm crystallite primary size and negative zeta potential. In the text, the nanoparticles were labelled as hNDs.

The FT-IR spectrum of hNDs was recorded using an IR Prestige-21 spectrometer (Shimadzu Europa GmbH, Düsseldorf, Germany) equipped with an ATR (Attenuated total reflectance) module. A few drops of hNDs were deposited on the ATR and were dried at room temperature to form a thin layer of hNDs. The measurements were carried out in the range of 400–4000 cm^−1^, using a ZnSe crystal with 0.2 cm^−1^ in resolution.

The surface chemical composition of the functionalised hNDs was investigated by X-ray Photoelectron Spectroscopy. The XPS measurements were performed using an ESCALAB 250Xi spectrometer (Thermo Fisher Scientific Ltd., Cambridge, UK), equipped with a mono-chromatic Al kα source, six channeltron detection system and a flood gun for charge neutralisation. A few drops of hNDs were deposited on a graded pure gold foil (99.9%) and were let to dry at room temperature. The binding energy scale was calibrated by positioning the C1s and Au4f_7/2_ peaks at 285.0 eV and 84.0 eV, respectively. All spectra were acquired and processed by Avantage v.5 software (Thermo Fisher Scientific Ltd., UK). More experimental details have been reported elsewhere [49].

Dynamic light scattering (DLS) and ζ-potential measurement were acquired at an angle of 173° with a Zetasizer Nano ZS90 instrument (Malvern, Herrenberg, Germany) equipped with a 633 nm He–Ne laser as previously described [50]. The measurements were performed at a fixed position (4.65 mm) with an automatic attenuator and temperature of 23 °C. Before measurements, the hND suspensions (2.5 mg/mL) were bath-sonicated for 30 min at 4 °C. Solvent-resistant micro cuvettes (ZEN0040, Malvern, Herrenberg, Germany) were used for experiments with a sample volume of 40 μL. The hydrodynamic diameter values were calculated by averaging the number-weighted diameter distribution measured with DLS. The ζ-potential of hNDs was calculated from the electrophoretic mobility using the Henry correction to Smoluchowski’s equation, implemented in the Zetasizer data-analysis software.

For the experiments with algal cultures, aliquots of the original hND colloidal suspensions (1% *w*/*v*) were diluted with milli-Q water to a concentration of 5 mg hNDs/mL. The diluted hNDs were autoclaved using a standard procedure (20 min, 121 °C, 1 bar) and bath-sonicated before use. All procedures were performed under sterile conditions to avoid alteration in algal growth and photosynthetic performance due to contaminations.

### 4.2. Algal Strain and Growth Conditions

A cell wall deficient strain of *Chlamydomonas reinhardtii*, CC-400, was purchased from the Chlamydomonas Resource Centre, University of Minnesota, USA. Algae were grown photoheterotrophically in 100 mL Tris-Acetate-Phosphate (TAP) medium [51] in 250 mL Erlenmeyer flasks at 24 ± 1 °C under continuous illumination, photosynthetic photon flux density (PPFD) of 50 µmol/m^2^/s and shaking at 150 rpm. Culture growth was registered over 72 h by measuring cell number per mL (Automated Cell Counter, TC10, Bio-Rad Laboratories, Inc, CA, USA), optical density at 750 nm (OD_750_) and Chl content, as previously described [52]. All experiments were performed using cultures in the late exponential growth phase (OD_750_ = 0.70 ± 0.05).

### 4.3. Chl a Fluorescence Measurements

Light-induced Chl *a* fluorescence transients (OJIP transients) were recorded in algal suspensions using a Plant Efficiency Analyser (PEA, Hansatech Instruments Ltd., King’s Lynn, Norfolk, UK). The OJIP transients were induced after 15 min dark incubation with a 3 s saturating pulse of red light with a maximum at 650 nm and PPFD of 3500 µmol/m^2^/s. The F_V_/F_M_ ratio, an empirical fluorescence parameter well correlated with changes in PSII activity during photoinhibition [39,53], was calculated from the fluorescence transient according to [54]:

(1) $$F_{V}{/F}_{M}=(F_{M}-F_{0})/F_{M}$$

where F_0_ and F_M_ are the fluorescence level at 50 µs after the onset of the illumination and the maximum fluorescence yield, respectively.

Where indicated, the F_V_/F_M_ parameter was also measured via the pulse-amplitude modulated method using a PAM-2000 fluorometer (PAM, Heinz Walz GmbH, Effeltrich, Germany) on 15 min dark-adapted samples. The F_0_ was excited by a train of weak probe flashes of 3 μs of red light (λ = 655 nm) at a frequency of 0.6 kHz. A saturating pulse of 0.8 s white light (λ < 710 nm, PPFD 6000 μmol/m^2^/s) generated by a 20 W halogen lamp was applied to induce the F_M_. The Chl *a* fluorescence was recorded through the wall of 10 mL glass flasks containing 5 mL algal suspension.

### 4.4. Assessment of hND Phytotoxicity

The phytotoxicity of the selected hNDs was tested in short-term (5 h) and long-term (72 h) exposure experiments. To this end, algal cultures were supplemented with different amounts of hNDs to obtain samples with 5, 10, 20, 40, 60, and 80 µg hNDs/mL final concentration and were grown under control conditions (see Section 4.2 of Materials and Methods). Short-term phytotoxicity tests were performed on cultures containing 10.4 ± 0.8 µg Chl/mL and (5.2 ± 0.2) × 10^6^ cells/mL and resemble the conditions of “immediate effect” experiments, while long-termed tests used cultures containing 5.1 ± 0.3 µg Chl/mL and (3.3 ± 0.1) × 10^6^ cells/mL similar to the “24 h pre-incubation effect” experiments (see Section 4.5 of Materials and Methods).

### 4.5. Assessment of hND Antioxidant Capacity in C. reinhardtii Cultures

The capacity of the hNDs to protect algal cells against oxidative stress was tested under two distinct conditions associated with the generation of ROS. Oxidative stress conditions were engendered using ROS-generating substances, such as MV or by subjecting the cultures to HL conditions.

#### 4.5.1. MV-Induced Oxidative Stress

The optimum MV concentration necessary to induce oxidative stress under our experimental conditions was identified in an independent set of experiments. To this end, cultures containing 10.4 ± 0.8 µg Chl/mL and (5.2 ± 0.2) × 10^6^ cells/mL were supplemented with MV to obtain 1, 2, 3, and 5 µM final concentration. The samples were exposed to growth conditions, and the MV-induced inhibition of the PSII photochemistry was evaluated by recording the F_V_/F_M_ parameter every hour over a period of 4.5 h. These preliminary experiments pointed out the 3 µM MV treatment as the one causing approximately 50% decrease of the F_V_/F_M_ parameter for the duration of the treatment (Supplementary Figure S4) and were used to induce conditions of oxidative stress in *Chlamydomonas* cultures.

In all MV-related trials, the potential antioxidant capacity of selected hNDs was evaluated by considering two different experimental protocols. In the first set of experiments (“immediate effect”), 3 µM MV and different amounts of hNDs were added simultaneously to Chlamydomonas cultures, and the samples were exposed to control growth conditions (PPFD 50 µmol/m^2^/s and 150 rpm shaking) for 4.5 h. In the second set of experiments (“24 h pre-incubation effect”), the cultures were initially incubated for 24 h with different amounts of hNDs at PPFD 10 µmol/m^2^/s and 150 rpm agitation. After this pre-incubation, the cells were collected by weak centrifugation (3 min at 2500× *g*, 22 °C), resuspended in ND-free growth medium and treated with 3 µM MV as in the “immediate effect” experiments.

To allow the comparison of the results obtained in the different treatments, all trials were performed using cultures standardised in terms of Chl content and cell number/mL, as follows: (i) all pre-treatments with hNDs were performed on cultures containing 5.1 ± 0.3 µg Chl/mL and (3.3 ± 0.1) × 10^6^ cells/mL; (ii) all MV treatments were performed on cultures containing 10.4 ± 0.8 µg Chl/mL and (5.2 ± 0.2) × 10^6^ cells/mL. The changes in the photochemical activity of PSII were evaluated by measuring the F_V_/F_M_ parameter.

#### 4.5.2. HL-Induced Oxidative Stress

The optimum PPFD necessary to provoke oxidative stress and the related photoinhibition under our experimental conditions was identified in an independent set of experiments. To this end, cultures containing 10.4 ± 0.8 µg Chl/mL and (5.2 ± 0.2) × 10^6^ cells/mL were exposed to 500, 1000, and 1500 µmol/m^2^/s and the F_V_/F_M_ parameter of the samples was recorded over a period of 4 h using PAM fluorometer. These preliminary trials indicated PPFD 1000 µmol/m^2^/s as suitable light intensity to induce approximately 50% reduction of F_V_/F_M_ ratio (Supplementary Figure S5) and were used to induce conditions of oxidative stress in *Chlamydomonas* cultures.

The potential of the hNDs to protect algal photosynthetic reactions against HL-induced oxidative stress was tested on cells pre-incubated for 24 h with hNDs as described in Section 4.5.1 (“24 h pre-incubation effect”). Cultures containing 5 µg Chl/mL were supplemented with 40 µg hNDs/mL and grown for 24 h at PPFD 10 µmol/m^2^/s and 150 rpm agitation. Then the cells were collected by weak centrifugation (3 min at 2500× *g*, 22 °C), resuspended in hND-free growth medium to 10 µg Chl/mL, and exposed to PPFD of 1000 µmol/m^2^/s for the HL treatment. The changes in the photosynthetic activity were evaluated by measuring the F_V_/F_M_ parameter using a PAM fluorometer.

Where indicated, the effect of the hNDs on the level of PSII photoinhibition under conditions of blocked de novo synthesis of PSII reaction centre D1 protein was evaluated on samples supplied with 400 μg/mL lincomycin (Sigma-Aldrich, Saint Louis, MO, USA).

### 4.6. Oxygen Evolution and Dark Respiration Measurements

The rates of O_2_ evolution and respiration were measured with a Clark-type oxygen electrode (Hansatech Instruments, King’s Lynn, Norfolk, UK) at 24 °C and continuous stirring on samples containing 10 µg Chl/mL. The rate of oxygen consumption of the cells in the dark (respiration rate) was measured after 3 min of dark adaptation. Light-induced oxygen evolution was measured under PPFD 1500 µmol/m^2^/s in the presence of 2 mM NaHCO_3_ as an additional carbon source.

### 4.7. Lipid Peroxidation

Lipid peroxidation of the algae was examined by determining the malondialdehyde (MDA) content [55]. Algal cells (25 mL) were collected by centrifugation (3000× *g*, 3 min) and stored at −80 °C before the analyses. The samples were homogenised in 5 mL of 10% trichloroacetic acid, and 1 mL of the homogenate was mixed with 2 mL of 0.5% thiobarbituric acid (Sigma-Aldrich) in 10% trichloroacetic acid (Sigma-Aldrich). The mixture was then incubated in a water bath at 95 °C for 40 min, cooled down to room temperature, and centrifuged at 4000× *g* for 15 min. The absorbance of the supernatant was recorded at 532 and 600 nm [56], and MDA content was calculated using an extinction coefficient of 155 mM^−1^ [57]. The results were expressed as µmol MDA per unit of dry weight.

Dry weight was determined using cells collected on glass microfibre filters (0.7 μm, GVS, USA) that were dried in the dehydrator at 90 °C until constant weight.

### 4.8. Ferric Reducing Antioxidant Power (FRAP) Assay

The FRAP assay was used to measure the total reducing power of the samples, which is associated with the antioxidant activity of biological specimens [58]. Cells were collected by centrifugation (3000× *g*, 3 min) and stored at −80 °C before the analyses. The samples were homogenised in trichloroacetic acid to break down the cellular wall, and 25 µL of cell extracts were mixed with 175 µL of distilled water and 1.5 mL of FRAP solution. FRAP reagent was prepared by mixing 2.5 mL of FeCl_3_·6H_2_O, 25 mL of acetate buffer (pH 3.6), and 2.5 mL of TPTZ solution (10 mM 2,4,6-Tris(2-pyridyl)-s-triazine dissolved in 40 mM HCl). The mixture was incubated at 37 °C for 10 min, cooled down to room temperature and its absorbance at 593 nm was recorded. The results were expressed as mmol Fe(II) equivalent per gram of dry weight.

### 4.9. Detection of Singlet Oxygen Production

^1^O_2_ generation was measured as a rate of histidine (His)-mediated oxygen uptake as previously published [59,60]. Algal samples containing 10 µg Chl/mL were adapted for 3 min in the dark with and without 5 mM His. Then the rate of O_2_ evolution was measured under PPFD 3000 µmol/m^2^/s at 24 °C using a Clark-type oxygen electrode (Hansatech Instruments, King’s Lynn, Norfolk, UK). ^1^O_2_ production rate was calculated as a difference of the O_2_ evolution rate between samples with and without His.

### 4.10. Statistical Analyses

Three biological replicates and 2–3 technical repetitions were performed throughout the experiments. The effects of different treatments were analysed using one-way ANOVA, and means were analysed using the Tukey test. The differences at *p* < 0.05 were considered statistically significant.

## Figures and Tables

**Table 1:** MDA level and FRAP value in *C. reinhardtii* cultures pre-incubated with 40 µg hNDs/mL for 24 h and in control cells exposed to 3 µM MV. Means of 2 independent experiments with 3 biological replicates (±SD, *n* = 6).

Sample	MDA µmol/g DW		FRAP mmol Fe(II)eq/g DW	
	CTR	NDs	CTR	NDs
T0	1.8 ± 0.2	1.7 ± 0.1	0.20 ± 0.01	0.20 ± 0.02
T3	3.1 ± 0.3 *	2.6 ± 0.3 *	0.36 ± 0.04 *	0.31 ± 0.04 *

* indicates statistically significant differences between T0 (before the addition of MV) and T3 (after 3 h of incubation with MV) at *p* < 0.05; MDA, malondialdehyde, FRAP, ferric-reducing antioxidant power.

**Table 2:** High light-induced changes in the O_2_ evolution and respiration rate of *C. reinhardtii* cultures pre-incubated with 40 µg hNDs/mL for 24 h and in the control cells. Respiration rate and light-induced O_2_ evolution were measured after 3 min of dark adaptation and under PPFD of 1500 µmol/m^2^/s, respectively, in the presence of 2 mM NaHCO_3_. Means of 2 independent experiments with 3 biological replicates (±SD, *n* = 6).

Sample	O_2_ Evolution Rate µmol O_2_/mg Chl/h		Respiration Rate µmol O_2_/mg Chl/h	
	CTR	NDs	CTR	NDs
T0	176 ± 8	168 ± 14	18 ± 0.7	18 ± 1.1
T2	101 ± 15 *	120 ± 11 *	40 ± 1.1 *^,b^	35 ± 1.5 *^,a^

* indicates a statistically significant difference between T0 and T2, and small caps letters indicate a significant difference between CTR and sND samples at *p* < 0.05.

**Table 3:** MDA level, FRAP value, and ^1^O_2_ production rate in *C. reinhardtii* cultures pre-incubated with 40 µg hNDs/mL for 24 h and in control cells exposed to 1000 µmol/m^2^/s for 2 h. Means of 2 independent experiments with 3 biological replicates (±SD, *n* = 6).

Sample	MDA µmol/g DW		FRAP mmol Fe(II)eq/g DW		^1^O_2_ Production Rate µmol ^1^O_2_/mg Chl/h	
	CTR	NDs	CTR	NDs	CTR	NDs
T0	1.8 ± 0.2	1.7 ± 0.1	0.20 ± 0.01	0.20 ± 0.02	16 ± 3	12 ± 2
T2	2.3 ± 0.4	2.0 ± 0.3	0.24 ± 0.01	0.23 ± 0.03	26 ± 3 *	20 ± 4 *

* indicate a statistically significant difference between T0 (before HL exposure) and T2 (after 2 h at 1000 µmol/m^2^/s) at *p* < 0.05; MDA, malondialdehyde, FRAP, ferric reducing antioxidant power.

## Data Availability

All data generated or analysed during this study are available from the corresponding author upon request.

## Supplementary Materials

The supporting information can be downloaded at: https://www.mdpi.com/article/10.3390/ijms24065615/s1.
