# Supplementary material for: Nanodiamond Particles Reduce Oxidative Stress Induced by Methyl Viologen and High Light in the Green Alga Chlamydomonas reinhardtii
Source: Int J Mol Sci. 2023 Mar 15;24(6):5615. doi: 10.3390/ijms24065615 (PMC10052329; doi:10.3390/ijms24065615)
Supplement: Supplementary file 1 [file ijms-24-05615-s001.zip › ijms-2212136-supplementary.pdf]

Supplementary Information

Nanodiamond Particles Reduce Oxidative Stress Induced by Methyl Viologen and High Light in the Green Alga *Chlamydomonas reinhardtii*

*Taras K. Antal[1†], Alena A. Volgusheva[2†], Adil A. Baizhumanov[2], Galina P. Kukarskikh[2], Alessio Mezzi[3], Daniela Caschera[3], Gabriele Ciasca[4], Maya D. Lambreva[5]**

[1]Laboratory of integrated ecological research, Pskov State University, 180000 Pskov, Russia

[2]Department of Biophysics, Faculty of Biology, Lomonosov Moscow State University, 119991 Moscow, Russia

[3]Institute for the Study of Nanostructured Materials, National Research Council, 00015 Monterotondo Stazione (RM), Italy

[4]Dipartimento di Neuroscienze, Università Cattolica del Sacro Cuore, Fondazione Policlinico Universitario "A. Gemelli", IRCSS, 00168 Rome, Italy

[5]Institute for Biological Systems, National Research Council, 00015 Monterotondo Stazione (RM), Italy

[†]These authors contributed equally to this work.

Correspondence to: Maya D. Lambreva, Institute for Biological Systems, National Research Council, Via Salaria Km 29,300, Monterotondo Stazione (RM) 00015, Italy, e-*mail:* *maya.lambreva@cnr.it*, ORCID ID: 0000-0001-5750-0899

Table S1. Binding Energy (BE) and XPS quantification (atomic %) of the hNDs sample.

Name	Peak BE	FWHM eV	Area (P) CPS.eV	Atomic %	assignment
C1s - 1	285.0	2.40	30075.30	73.4	C – C
C1s - 2	286.6	2.40	5584.43	13.6	C – O
N1s	398.8	2.86	1294.65	1.8	Amine
Na1s	1071.3	3.06	1541.18	0.6	Na^{+}
O1s - 1	531.1	2.70	8945.43	8.0	C – O, –OH
O1s - 2	529.2	2.70	2442.41	2.2	ZrO_x
Zr3d5	181.2	2.14	793.94	0.5	ZrO_x

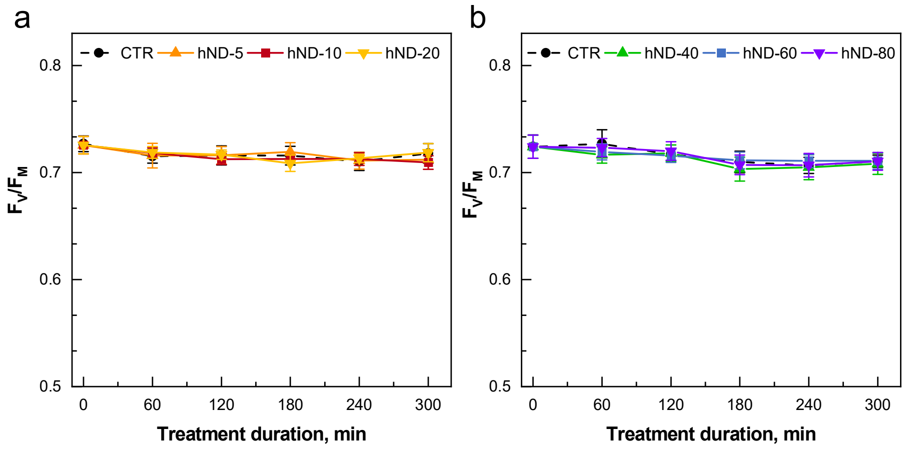

Figure S1. Short-term effects of low (**a**) and high (**b**) concentrations of hNDs on the Chl *a* fluorescence parameter F_V/F_M of *Chlamydomonas reinhardtii* cells. Algal cultures containing 10.2±0.4 µg Chl/mL were supplemented with 5, 10 or 20 µg hNDs/mL (low concentration) or 40, 60 or 80 µg hNDs/mL (high concentration) and the changes in PSII activity were followed for 5 h. Means of 3 biological replicates (±SD, *n*=3).

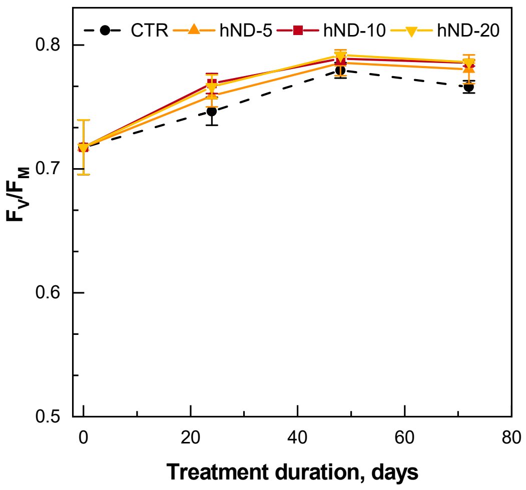

Figure S2. Long-term toxicity test of low hND concentrations in *C. reinhardtii* cells. Algal cultures containing 5.1±0.3 µg Chl/mL were supplemented with 5, 10 or 20 µg hNDs/mL and PSII activity was followed for 72 h. Means of 3 biological replicates (±SD, *n*=3).

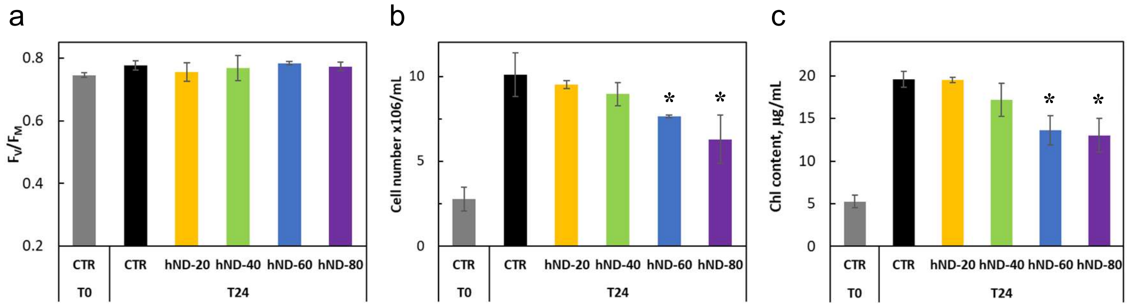

Figure S3. Effect of 24 h pre-incubation of *C. reinhardtii* cultures with different concentrations of hNDs (20, 40, 60 and 80 µg/mL) on PSII photochemistry (**a**), cell number per mL (**b**) and Chl content (**c**). CTR T0 point indicates the parameters of the initial cultures exploited in the "*24 h pre-incubation*" experiments; T24 points characterise the cultures used in the experiments shown in Figure 4b and Figure 5b of the main text. Asterisks indicate statistically significant difference between the T24 CRT and hND samples at P<0.05. Means of 2-3 independent experiments with 2-3 internal replicates (±SD, *n*=4-9).

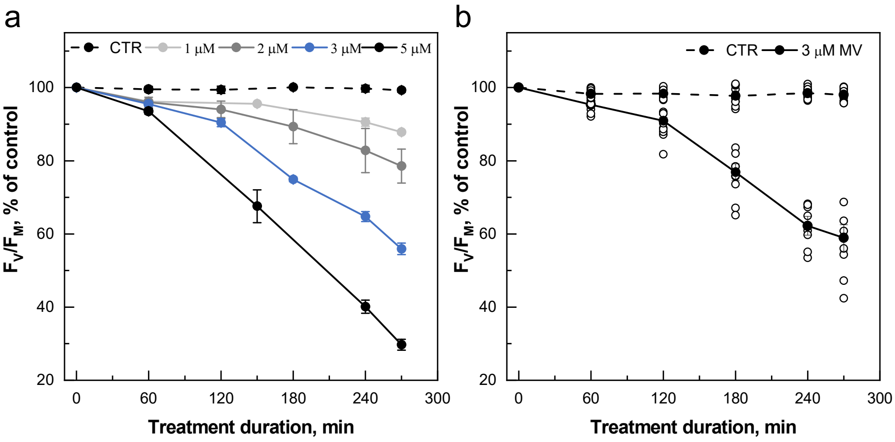

Figure S4. (**a**) Inhibition of PSII photochemistry (F_V/F_M parameter) in *C. reinhardtii* cultures treated with different concentrations of methyl viologen (MV) at a PPFD of 50 µmol/m^2/s. Values are presented as % of the control at T0, F_V/F_M(T0) = 0.73±0.006; mean of 3 biological replicates (±SD, n=3). (**b**) Variability of F_V/F_M parameter in control and 3 µM MV treated samples. Values are expressed as % of the T0 values, F_V/F_M(T0) = 0.72±0.004 and F_V/F_M(T0) = 0.73±0.006 in the MV and CTR samples, respectively. Empty circles represent single experimental values, black circles represent mean values. Means of 10-12 independent experiments with 2-3 biological replicates (±SE, *n*=10-12).

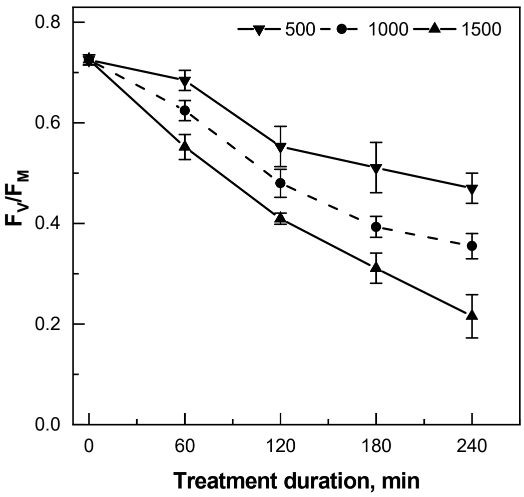

Figure S5. Changes in the F_V/F_M parameter of *C. reinhardtii* cultures exposed to different PPFDs. Cultures containing 10 µg Chl/mL were exposed to 500, 1000 or 1500 µmol/m^2/s and F_V/F_M was measured every hour for the following 4 h. Means of 4 independent experiments with 3 biological replicates (±SD, *n*=12).
